# The role of stigma in cannabis use disclosure: an exploratory study

**DOI:** 10.1186/s12954-024-00929-8

**Published:** 2024-01-26

**Authors:** Daniel D. King, Christopher J. Gill, Carey S. Cadieux, Neha Singh

**Affiliations:** 1 College of Nursing, Rosalind Franklin University of Medicine and Science, 3333 Green Bay Road, North Chicago, IL 60064 USA; 2 National Board of Certification and Recertification for Nurse Anesthetists, 8725 West Higgins Road, Suite 525, Chicago, IL 60631 USA; 3 Pacific College of Health and Science, 7445 Mission Valley Road #105, San Diego, CA 92108 USA; 4 Bouvé College of Health Sciences, School of Nursing, Northeastern University, 360 Huntington Avenue, Boston, MA 02115 USA

**Keywords:** Cannabis, Stigma, Disclosure

## Abstract

**Background:**

Although cannabis use incidence, societal acceptance, and legislation all trend positively, cannabis remains federally illegal in the USA. Prior studies have revealed that patients are reluctant to disclose their cannabis use history in the healthcare system, which can negatively impact patient care. This study reports the frequency of cannabis use disclosure with special considerations for stigmatization. To better understand the limitations, providers face in providing collaborative, comprehensive, and informed care, this study evaluated four domains of stigma: perceived, anticipated, enacted, and internalized.

**Methods:**

This study used a descriptive exploratory design. Data collection occurred using an anonymous, online national survey with a convenience sample in the USA. Recruitment relied on electronic media and occurred between July and December 2022. Participants were adults older than 21 years and self-identified as having used cannabis and accessed the healthcare system within the last five years. The survey measured demographic characteristics, cannabis use, and disclosure patterns. Stigma was measured using the Stigma Use Stigma Mechanism Scale (SU-SMS) and Substance Abuse Use Self-Stigma Scale (SASSS) with language modifications for cannabis. Ordinal logistic regression models were performed to evaluate associations between the frequency of cannabis use disclosure patterns and each stigma category. Associations were assessed using Chi-squared or Fisher’s exact tests.

**Results:**

Data were available for 249 respondents. Most participants (57.1%) reported initiating a conversation about cannabis with their healthcare provider; 27.8% of the time, cannabis is never discussed, and healthcare providers initiate only 15.1% of related discussions. Anticipated stigma [95% CI 1.045–1.164] and total stigma [95% CI 1.001–1.039] had statistically significant associations with nondisclosure. Annual household income (*p* = .04), chronicity of cannabis use (*p* = .03), frequency of cannabis use (*p* = .02), and a known amount of CBD in products consumed (*p* = .01) had statistically significant associations with the frequency of cannabis use disclosure.

**Conclusions:**

Patients who use cannabis experience stigmatization in the healthcare setting that may limit disclosure of cannabis use history. Future studies would be well served to explore anticipated stigma more deeply. Healthcare providers should be knowledgeable to lead such conversations relating to cannabis while maintaining an unbiased perspective.

**Supplementary Information:**

The online version contains supplementary material available at 10.1186/s12954-024-00929-8.

## Introduction

With increasing incidence, healthcare providers are identifying and caring for patients who use medicinal and recreational cannabis. According to the most recent United States (US) National Survey on Drug Use and Health, the annual percentage of cannabis use rose from 11 percent to 17.5 percent between 2002 and 2019, demonstrating that more than 48.2 million Americans aged 12 years or older currently use cannabis [1]. Accompanied legislative efforts, increased access, and growing social acceptance of use are among the significant contributors to this shift [2–4]. At the time of this article’s publication, thirteen states have pending recreational measures on their legislative agendas, further adding to most of the USA wherein cannabis has been decriminalized or legalized for recreational and medical uses. Most Americans demonstrate positive trends toward acceptance of legalization amidst mounting use [3]. For the first time in modern history, the number of cannabis users in the USA has surpassed the number of those using tobacco products, according to a recent *Gallup* poll [5].

Patients utilize cannabis for many reasons that may be largely unknown to healthcare providers or poorly understood. Several perceived benefits of cannabis include anxiolytic and analgesic properties and demonstrated effectiveness as a neuroleptic agent in treating conditions such as muscle spasticity or seizure disorders [6]. Cannabis has been evidentially affirmed to have immunosuppressive and antineoplastic properties, deeming its utility for cancer patients for prophylaxis and treatment of chemotherapy-induced nausea and vomiting and appetite modulation [7]. Among several qualifying conditions for medical prescribing of cannabis, chronic pain remains by far the most common, followed by multiple sclerosis, for which both possess substantial evidence of efficacy [6, 7]. Additional conditions commonly cited include glaucoma, HIV/AIDS, and post-traumatic stress disorder, among others [6–8]. Yet, for most Western medical practitioners, cannabis is not traditionally considered a medication nor influences routine prescribing practices for such conditions [8]. This may be due to subjective norms or known or perceived risks [6, 9]. In such cases, patients may self-substitute conventional medicines with cannabis to treat their conditions [10, 11]. A recent systematic review and meta-analysis found that the pooled prevalence for substituting medicinal cannabis drugs is 60%, with narcotics/opioids, anxiolytics, and antidepressants being the most commonly replaced [11].

Despite fostering social acceptance through successful legislative efforts, cannabis users have persistently demonstrated reluctance to disclose their associated use behaviors and patterns with healthcare providers [12]. The proposed rationale includes bias, stigma, fear of consequences, and undetermined significance of disclosure [4]. Furthermore, caregivers lack knowledge of the human endocannabinoid system in light of relevant clinical implications, nor do they readily possess formal education to navigate conversations regarding cannabis use with patients effectively [8, 13, 14]. Due to a lack of regulatory standards, among other concerns, cannabis is often not included in medication reconciliation records, further decreasing routine awareness [15]. Such deficiencies ultimately impair the provider’s decision-making ability since it is not based on comprehensive data or the holistic view of the patient. Consequently, cannabis users are predisposed to a higher risk for poor outcomes such as drug–drug interactions, poor response to anesthesia, higher pain medication doses in the postoperative period, or worsened disease states as seen in other stigmatized conditions [16–19].

### Literature review

A PubMed, EBSCO, and CDC database literature review explored stigmatization related to cannabis disclosure and behavioral patterns in healthcare. Inclusion criteria comprised full-text, English articles from the past decade, and pivotal publications predating 2012. Screening involved titles, abstracts, and reference lists, with keyword identification. Synthesis and study comparison were facilitated using an evidence grid, revealing common themes on the medical provider and patient perceptions and stigma.

Patients, particularly those with chronic conditions like refractory pain or seizure disorders, seek cannabis for its proposed benefits [20, 21]. However, divergent perceptions of cannabis efficacy exist among patients, nurses, and physicians. HaGani et al. [22] found that young patients had positive views, whereas physicians, especially compared to nurses, held fewer positive attitudes toward medicinal cannabis [22]. Clinicians with an oncology background exhibited more positive perceptions, likely due to the drug’s sought-after antineoplastic and immunosuppressive effects. [22]. Negative perceptions are more likely for stigmatized conditions like chronic pain or mental illnesses when compared to others, like cancer [23]. Increased provider knowledge and education about cannabis were associated with positive perceptions [22].

The literature consistently reveals discordant opinions between patients and healthcare providers on cannabis [11, 14, 21, 22]. While patients favor cannabis for conditions like pain and anxiety, clinicians often prefer traditional treatments, including opioid-based therapies [20, 21, 24]. In Massachusetts, a study of musculoskeletal trauma patients showed that 81% believed in cannabis as a medication, with 13.1% using it during recovery, reporting pain reduction and decreased opioid use [21]. In rheumatoid disease, Gavigan et al. [10] found that 89% of patients tried cannabinoids, but most providers did not consider cannabis a treatment modality. Similar discrepancies were noted in a study of rheumatology patients in Great Britain, where despite patients experiencing pain relief with cannabis, 78% of practitioners did not find it helpful [25]. This divergence, along with knowledge gaps, contributes to stigmatization and reluctance to disclose cannabis use, hindering patient self-efficacy [25, 26].

Mistrust, stigmatization, and reluctance to disclose cannabis use pose barriers to fully informed care, emphasizing the importance of comprehensive health assessments inclusive of cannabis use history [12]. Discrepancies between disclosed and actual cannabis use indicate potential provider failure to assess accurately and patients’ reluctance to disclose [12]. Stigmatization, a likely reason for underreporting, aligns with historical trends and societal factors [27, 28]. Recognizing value in patient-reported data, deficiencies may lead to worsened outcomes such as suboptimal dosing in the perioperative setting [12, 17, 18, 29].

### Theoretical framework: stigma

In 1963, Canadian-American sociologist Erving Goffman denoted the term *stigma* as having Greek origins “to refer to bodily signs designed to expose something unusual and bad about the moral status of the signifier.”[30 (p1)] Stigma is the negative attitude held toward an individual or group due to specific characteristics or attributes, such as those prevalent regarding cannabis use patterns [31]. Proficient use of the term led Goffman to become a prominent developer of labeling theory. To build upon the work of Howard Becker, this theory reveals that over time, affected individuals begin to self-identify as deviants from cultural norms and display behaviors that reflect the terminology or labels others have placed on them [4, 28, 31, 32]. Cannabis use can be stigmatized as deviant behavior similar to that observed in criminology, tied to societal labeling, discrimination, and devaluation [4, 33]. Distinctively, discrimination involves unfair or unequal treatment, whereas devaluation involves denying the significance of the individual [34]. In his work, Goffman further described stigma as having profound social consequences, impacting personal identity, and leading to the questioning of oneself [30]. Stigma has well-known, deleterious health consequences influenced by widening health disparities, precluding relationships, and magnifying negative psychosocial experiences [16].

### Stigma domains

Several domains, or dimensions, of stigma exist to reflect that the wide-ranging course is experientially complex and multifaceted. We sought to elicit whether certain stigmatization experiences were significant in cannabis users as they present in US healthcare settings. Thus, we primarily characterized stigmatization for this study using four domains: (1) perceived, (2) internalized, (3) anticipated, and (4) enacted stigma.

#### Perceived stigma

Perceived stigma involves an individual’s cognizance and perception of negative attitudes, concepts, or societal beliefs about a particular group [33, 35, 36]. Within the perceived stigma domain, an affected individual may demonstrate elevated awareness of how cannabis users may be treated in the healthcare system. Subcomponents of perceived sigma include perceived devaluation and discrimination and may be combined with additional factors, such as labeling [37]. Perceived stigma is a critical dimension in cannabis stigmatization research for which there is believed to be great potential to lead a change movement [38]. This potential involves the recognition of significantly deleterious impacts on the provider–patient relationship with negative psychological consequences, inconsistent or inaccurate treatment regimens, and worsened medical outcomes. Perceived stigma has also been interpreted to mean the fear of enacted stigma [31, 39]. This leads to reluctance to seek healthcare services, topic avoidance, and impaired feelings of self-worth [27, 28, 40].

#### Enacted stigma

Stereotyping leads to definitive actions against a social typecast in the enacted stigma domain [41]. Enacted stigma is that which is experienced and refers to overt actions that demonstrate discrimination or devaluing behavior [35, 42]. For example, cannabis users may be readily dismissed, denied services, have appointments cut short, or have poorer outcomes when compared to non-users [43]. Family, friends, and healthcare workers can be sources of enacted stigma. The resulting lived experiences shape the cannabis user’s future decision-making and self-devaluation.

#### Internalized stigma

Internalized stigma is also called self or felt stigma and arrives at the level of an individual person [44]. Rather than a heightened awareness of surrounding stigma, in this case, detected stereotypes about a group have become internalized, accepted, and embedded in the individual [35, 44]. This highly depends on how others view the cannabis user’s ability to perform conventional roles at home and work [4]. The contemporary place of cannabis in society, its popularity, known health benefits or harms, and legislative status are all necessary drivers. Interestingly, a recent study evaluating the impact of social media discussions found internalized stigma the most common, with 38% of cannabis-related posts involving the domain [45]. Internalized stigma has been studied among cannabis users and demonstrated to shape their attitudes, beliefs, and behaviors [4]. Hathaway et al. [4] identified internalizing stigma patterns in cannabis users who described fears of being caught, changing cannabis use behaviors around others, and hiding use to conform to the believed expectations of others.

#### Anticipated stigma

Anticipated stigma refers to specific beliefs about expected events, scenarios, or situations to unfold in the future based on stigmatization [35, 46]. For example, anticipated stigma within the cannabis user could involve believing that a practitioner will devalue them if they disclose their use and behavior patterns [41]. Experiencers of anticipated stigma often spend excessive energy worrying about what others may think about them [45]. Aside from worsened mental health, anticipated stigma has been correlated with worsened physical health outcomes, as correlated with CD4 count in HIV patients in a prior study, as one example [46].

### The stigmatization experience

Legislative changes and evolving societal norms have led to regional variations in the stigma associated with cannabis use, with stricter policies correlating with increased stigma, particularly in terms of devaluation [33]. Cannabis stigma is linked to adverse mental and physical health outcomes [43]. Within healthcare, users fear disbelief in their medical reasons for cannabis use, impacting emotional well-being and therapeutic benefits. Despite societal acceptance, fear of judgment remains a significant barrier to sharing cannabis use details with caregivers [40]. Bottorff et al.’s qualitative research underscores cannabis as a stigmatized medicine, especially for conditions like HIV/AIDS, pain disorders such as fibromyalgia, and mental health disorders, including anxiety [28, 47]. The legal status of cannabis intertwines with perceived stigma, influencing patient disclosure and leading to internalized stigma, concealment, fear, and distancing behaviors [4, 33].

In a qualitative study of Baby Boomers in the San Francisco Bay area, despite cannabis normalization, nearly half of the participants preferred nondisclosure due to judgment concerns [27]. With perceived threats or harms to well-being, participants adopted strategies to minimize risks, such as private use and odor-masking practices, preserving identity without stereotyping. Distinguishing legal risks from stigma is also crucial, as perceived legal ramifications deter disclosure within the provider–patient relationship [15, 27]. Legal status significantly influences stigmatizing behaviors, contributing to regional variations in perceived stigma and provider biases. In regions with greater legal consequences, patients are more likely to experience stigma and adopt harm-reducing behaviors [33].

### Purpose

This study aimed to assess rates and associations of cannabis use disclosure and discussion with consideration for stigmatization as patients in the US healthcare system. To better understand cannabis stigmatization experiences in the US healthcare setting, mainly related to disclosure and assessment ability, a foreground question among the researchers was asked as an etiology question to know “to what extent a factor, process, or condition is highly associated with an outcome, usually undesirable” [48 (p39)]. Specifically, we asked to what extent stigma domains are experienced by cannabis users aged 21 years and older when presenting as patients in the US healthcare system within the last five years, with consideration for subject demographics, disclosure of use, and cannabis use patterns. A national (US) descriptive exploratory survey design was the most appropriate method to answer this question. The hypotheses were as follows:

1. Cannabis users experience stigma in healthcare settings.
2. Cannabis users who present as patients in the healthcare system are reluctant or less likely to disclose cannabis use due to stigmatization.
3. The frequency of cannabis use disclosure is increased in geographic regions where legalization has been established for recreation and medicinal use.
4. The frequency of cannabis use disclosure increases in persons with greater frequency and longer duration of cannabis use.

## Methods

Prior approval for this descriptive exploratory study to be conducted with exempt status was obtained from the Northeastern University Institutional Review Board (IRB# 22-06-17). A randomized sample was collected via a web-based survey of cannabis users in the USA. An electronic survey link was distributed utilizing outlets such as related societal organization newsletters, email, and social media posts. Leaf411™ was a primary survey distributor. Leaf411™ is a 501(c)(3) nonprofit operating a cannabis-trained nurse guidance line. Additional survey distributors included the American Cannabis Nurses Association (ACNA), TheAnswerPage.com™, and Holistic Caring®. The survey was only completed once voluntarily by anonymized study subjects with no further follow-up. The authors had no direct interaction with the subjects. To incentivize the study, upon completion, all respondents received a promotional code for nurse consultation and commercial products in partnership with Leaf411™.

In a self-administered electronic questionnaire, the data collected included:

- Demographics deemed necessary as potentially relating to stigmatized persons
- Cannabis use characteristics including route, dose, frequency, and chronicity
- Reasons for cannabis use
- Cannabis use disclosure patterns in the health care setting including frequency of occurrence, indications for the desire to disclose, and initiator of discussion between patient and provider
- Perceived, anticipated, internalized, enacted, and total stigma scores using adapted versions of the Substance Use Stigma Mechanisms Scale (SU-SMS) and Substance Abuse Self-Stigma Scale (SASSS)

The full survey is included in Additional file 1. Survey data were collected and managed using the Qualtrics survey platform. Eligibility criteria for this study were (a) adults aged 21 years or older who (b) used cannabis (marijuana) within the last five years and (c) received medical care within the previous five years.

An a priori analysis was conducted. Using prior literature, an odds ratio of 1.7 was selected, and there was found to be an 80% chance at a significance level of .05 to correctly reject the null hypothesis that a particular category of the primary predictor variable (frequency of cannabis use disclosure) is not associated with the value of the outcome variable (stigma type), with a total 492 participants. Prior publications validating the SU-SMS and SASSS scales generated sample population numbers of 178 and 352 participants, respectively [41, 49].

Descriptive statistics and frequency distributions were reported for categorical and continuous variables. Mean and standard deviations of continuous variables and frequencies and percentages of categorical variables were calculated. Associations between categorical variables were assessed using Chi-squared or Fisher’s exact tests. Ordinal logistic regression models were performed to evaluate the associations between frequency disclosure patterns and each stigma category (enacted, anticipated, perceived, internalized, and overall stigma). A regression analysis using a backward elimination method was performed to determine associations between the frequency of disclosure (always, sometimes, or never) with participant demographics as well as cannabis use and disclosure patterns and stigma scores. Interaction effects were used to assess the interaction between any stigma type and its association with disclosure patterns. A multivariate ordinal logistic regression model was created by adjusting for additional covariates. The backward elimination selection method was used to predict the best-fit model. A *p*-value of significance level < .05 was considered statistically significant. If the *p*-value of the feature was greater than the significance level (alpha = .05), the feature was removed. R^2 (percent of variation), adjusted *R*^2^, Cp Criterion, PRESS criterion, and lowest AIC (Akaike information criteria) were used to decide the best-fit model and the potential variables to be included in the model.

### Definition of primary and secondary outcomes

Primary outcomes for measurement were stigma scores and frequency of cannabis use disclosure. Secondary outcomes included frequency of cannabis use disclosure and demographic data, including the legal status of cannabis in the reported state of residence, as well as cannabis use characteristics.

### Measuring stigma

A secondary literature review was conducted utilizing PubMed and EBSCO to identify prior scientifically validated instruments that assess stigma. Findings were that tools commonly evaluated stigma in the setting of illicit drug use, substance use disorders, mental health, and HIV/AIDS. However, these authors identified no tool specific to cannabis stigma in the healthcare setting that could be readily adopted. By consensus, the Substance Use Stigma Mechanisms Scale (SU-SMS) and Substance Abuse Self-Stigma Scale (SASSS) were most amenable to the authors for adaptation and use in cannabis patients [41, 49].

#### Substance Use Stigma Mechanisms Scale (SU-SMS)

The Substance Use Stigma Mechanisms Scale (SU-SMS) was co-developed by Laramie R. Smith, Ph.D. and Valerie A. Earnshaw, Ph.D., using a stigma framework with intended use and adaptability for a broad range of substance-using populations [41]. The original survey consists of 18 items that examine enacted (6 items), anticipated (6 items), and internalized stigma (6 items). All responses are administered using a five-point Likert-style scale with higher scores indicating the greater intensity of stigma experience. An average score may be calculated for each category. A study utilizing the theory-based scale in 175 diverse, substance-using participants found high internal consistency across all scales ($\alpha \hspace{0.17em}$= .90–.93) and subscales ($\alpha \hspace{0.17em}$= .90–.95). Correlated error variance and confirmatory factor analysis supported high structural and construct validity. Additionally, the SU-SMS was found to have high levels of internal reliability and generalizability [41]. We included and adapted the SU-SMS with expressed permission as follows: (1) all six items from the enacted stigma subscale were included, and “family members” was replaced with “healthcare workers,” (2) all six items from the anticipated stigma subscale were included and “family members” replaced with “healthcare workers,” and (3) all six items from the internalized stigma subscale were included and “alcohol and/or drugs” replaced with “cannabis.”

#### Substance Use Self-Stigma Scale (SASSS)

The Substance Use Self-Stigma Scale (SASSS) was developed by Jason B. Luoma, Ph.D., to measure self-stigma in substance misuse [49]. The questionnaire consists of 40 items that appraise self-devaluation (8 items), fear of enacted stigma (9 items), and stigma avoidance and values disengagement (23 items). Items are measured on a five-point Likert-style scale, with higher scores indicating a more significant endorsement of stigma. In a subsequent study by Luoma et al. [49], items related to fear of enacted stigma were found to have high internal consistency ($\alpha$ = .88), reliability, and predictability for correlation. Through consensus, we included and adapted six of nine relevant items from the subscale for fear of enacted stigma, which essentially defines perceived stigma as the fear of being discriminated against. Survey language was adapted so that “people” was replaced with “healthcare workers” and “substance” was replaced with “cannabis.” Expressed permission was obtained for using the adapted SASSS in this study.

## Results

### Demographics

Of 266 respondents who commenced the survey between July and December 2022, 5 did not meet eligibility criteria and 12 did not proceed to the demographics page. Thus, 249 participants were included for data extraction. Participant demographics are displayed in Table 1. The mean (± standard deviation) age of participants was 50.2 ± 13.2 years, and most participants were female (70.7%, 176/249) and white (83.1%, 207/249). Self-reported nonwhite race categories included American Indian or Alaskan Native (2/249), Asian (2.4%, 6/249), and Black or African American (6.0%, 15/249). Fourteen participants self-identified with a different race category, which included the free-text entries: multiracial (2.0%, 5/249), Hispanic (1.2%, 3/249), Mediterranean (1/249), Native American (1/249), Semitic (2/249), and unknown (1/249). Most participants reported being married at the time of the survey (52.2%, 130/249). Because several states of residence were reported, data were collapsed into categories based on legality. Most participants reported residence within a state where cannabis is fully legalized for medicinal and recreational purposes (61%, 152/249), with California (15.3%, 38/249) and Colorado (11.2%, 28/249) being among the most significant two recorded. Most participants reported earning a bachelor’s degree or higher (63%, 157/249). Diversity in household income was reported, with most participants earning an average household income greater than $105,000 per year (35.7%, 89/249). Yet, the second most reported earning group was fewer than $35,000 annually (23.3%, 58/249).

**Table 1:** Demographic characteristics

Characteristic	
Age, *n* = 211	50.2 (± 13.2)
Gender, *n* = 249	
Female	176 (70.7%)
Male	68 (27.3%)
Transgender male	1 (.4%)
Prefer not to answer	4 (1.6%)
Race, *n* = 249	
White	207 (83.1%)
Black or African American	15 (6.0%)
Asian	6 (2.4%)
American Indiana or Alaskan Native	2 (.8%)
Other	14 (5.6%)
Prefer not to answer	5 (2.0%)
Cannabis legalization status in reported state of residence, *n* = 249	
Legalized	152 (61.0%)
Medical and decriminalized	36 (14.5%)
Medical	36 (14.5%)
CBD with THC as an ingredient only	13 (5.2%)
Decriminalized	5 (2.0%)
Fully illegal	3 (1.2%)
Outside United States	4 (1.6%)
Highest level of education, *n* = 249	
Less than bachelor’s degree	88 (35.3%)
Bachelor’s degree	84 (33.7%)
Greater than bachelor’s degree	73 (29.3%)
Prefer not to answer	4 (1.6%)
Annual household income, *n* = 249	
< $35,000	58 (23.3%)
$35,000 to less than $70,000	42 (16.9%)
$70,000 to less than $105,000	47 (18.9%)
> $105,000	89 (35.7%)
Prefer not to answer	13 (5.2%)
Marital status, *n* = 249	
Now married	130 (52.2%)
Separated/widowed/divorced	68 (27.3%)
Never married	44 (17.7%)
Prefer not to answer	7 (2.8%)

### Cannabis use characteristics

Self-reported cannabis use characteristics among the participants are reported in Table 2. Among these, the most prevalent, primary routes reported were smoking (42.6%, 106/249), followed by vaping (20.9%, 52/249), and the use of edibles (14%, 35/249). Uncategorized but self-reported “other” routes included dry herb vape and concentrate dabbing. Cannabis use was reported to occur on most (21 or greater) days of the month for 71% (176/249) of participants. Notably, one-third of participants (33.1%, 82/248) reported using cannabis greater than three times daily. The most significant proportion of those surveyed (48.4%, 120/248) had been using cannabis for over ten years. Most participants (78.7%, 196/249) reported knowing the amounts of CBD and THC in their consumed products. When known, the most frequently recorded average daily dose of CBD was said to be between 5 and 20 mg/day (54.4%, 106/195), and the most common range of THC dose used was more widely variable from one to more than 30 mg per day. Most participants (60.5%, 118/195) reported THC concentrations greater than 15% in their consumed products. The ten most frequently reported reasons for which the respondents used cannabis are reported in Table 3. Less frequent reasons included nausea/vomiting (44/249), appetite (42/249), autoimmune disease (30/249), neuromuscular disease (15/249), bowel disease (15, 249), cancer (13/249), seizure disorder (6/249), spinal cord disease (6/249), glaucoma (6/249), brain disorder (5/249), hepatitis (3/249), kidney disease (3/249), bladder disorder (1/249), and terminal illness (3/249). “Other” reported qualitative responses included: avoidance of nightmares and overactive thinking, motivation, and focus, attention-deficit/hyperactivity disorder, cognitive function, chronic pancreatitis, non-alcoholic steatohepatitis, Ehlers–Danlos syndrome, restless leg syndrome, irritable bowel syndrome and digestion, libido, menopause, and inflammation.

**Table 2:** Cannabis use characteristics

Route most often used, *n* = 249	
Smoking	106 (42.6%)
Vape	52 (20.9%)
Edible	35 (14.1%)
Tincture	21 (8.4%)
Oil	12 (4.8%)
Capsule	9 (3.6%)
Topical	5 (2.0%)
Lozenge	1 (.4%)
Other	8 (3.2%)
Known amount of CBD/THC in products normally consumed, *n* = 249	
Yes	196 (78.7%)
No	53 (21.3%)
Average daily amount of CBD normally consumed, *n* = 195	
5–20 mg	106 (54.4%)
21–29 mg	29 (14.9%)
> 30 mg	32 (16.4%)
Unknown	28 (14.4%)
Average daily amount of THC normally consumed, *n* = 195	
1–5 mg	39 (20.0%)
5–10 mg	29 (14.9%)
10–20 mg	37 (19.0%)
20–30 mg	35 (18.0%)
> 30 mg	33 (16.9%)
Unknown	22 (11.3%)
THC concentration normally consumed, *n* = 195	
< 2.49%	14 (7.2%)
2.5–9.99%	18 (9.2%)
10–14.99%	11 (5.6%)
15–19.99%	38 (19.5%)
20–24.99%	38 (19.5%)
> 25%	42 (21.5%)
Unknown	34 (17.4%)
Number of days cannabis used in last 30 days, *n* = 248	
1 day or less	12 (4.8%)
2–5 days	17 (6.9%)
5–10 days	21 (8.5%)
11–20 days	22 (8.9%)
21 days or more	176 (71.0%)
Frequency of cannabis use on days used, *n* = 248	
Once	66 (26.6%)
Twice	50 (20.2%)
Three times	42 (16.9%)
Greater than three times	82 (33.1%)
Unknown/do not recall	8 (3.2%)
Duration of cannabis use, *n* = 248	
Less than 1 year	20 (8.1%)
1–5 years	65 (26.2%)
5–10 years	43 (17.3%)
Greater than 10 years	120 (48.4%)

**Table 3:** Reasons for cannabis use (*N* = 249)

Rank	Reason	Frequency
1	Anxiety	161
2	Pain	157
3	Sleep	141
4	Depression	109
5	Recreation/leisure	88
6	Arthritis	73
7	Post-traumatic stress disorder	68
8	Muscle spasm	62
9	Headache/migraine	61
10	Neuropathy	49

### Cannabis use disclosure

Descriptive cannabis use disclosure patterns in the healthcare setting are described in Table 4. When discussing cannabis use with their healthcare providers, most reported making their cannabis use known in some capacity, with 77.5% (193/245) stating “sometimes” or “always.” The patient’s comfort level with their healthcare provider was the primary reason influencing disclosure (42.5%, 104/245). In most circumstances (57.1%, 140/245), the surveyed patient indicated leading discussion about cannabis use themselves, whereas only 11.4% (28/245) of the time healthcare providers inquired directly with the patient as an influencer of cannabis use disclosure. 27.8% (68/245) of the time, respondents indicated that the discussion around cannabis use was never initiated by either person.

**Table 4:** Cannabis use disclosure patterns in the healthcare setting

How often do you make your cannabis usage known to healthcare providers? (*n* = 245)	
Always	113 (46.1%)
Sometimes	80 (32.7%)
Never	52 (21.2%)
What most influences your desire to disclose your cannabis use? (*n* = 245)	
Healthcare provider asks	28 (11.4%)
Comfort level with healthcare provider	104 (42.5%)
Unknown	7 (2.9%)
I do not disclose my cannabis use	41 (16.7%)
Other	65 (26.5%)
When in the healthcare setting, who initiates discussion of your cannabis use? (*n* = 245)	
Myself	140 (57.1%)
Healthcare provider	37 (15.1%)
Neither myself nor healthcare provider	68 (27.8%)

Statistical analyses were performed to determine the significance of the frequency of cannabis use disclosure with demographic variables and cannabis use patterns (Table 5). Annual household income, chronicity, frequency of cannabis use, and the known average amount of CBD consumed daily were all determined to have statistically significant associations with the frequency of cannabis use disclosure. Those participants with annual incomes greater than $105,000 did not disclose cannabis use with their healthcare providers 54% of the time, *X*^2^(8, *N* = 211) = 16.5, *p* = .04. Frequency of disclosure was also highly associated with chronicity of use, *X*^2^(6, *N* = 211) = 13.6, *p* = .03 as well as the frequency of use on the number of days per month (*p* = .02). Personal knowledge of the amount of CBD contained in products consumed carried a statistically significant association with the frequency of disclosure* X*^2^(6, *N* = 211) = 16, *p* = .01. However, knowledge of the average amount of THC in consumed products was not associated with frequency of cannabis use disclosure. Age, gender, race, level of education, and marital status all had no statistically significant correlation. Interestingly, the legal status of cannabis in the reported state of residence had no statistically significant association with the frequency of cannabis use disclosure. Complete item analysis for frequency of cannabis use disclosure is included in Additional file 2.

**Table 5:** Significance of Frequency of Cannabis Use Disclosure by Variable of Interest

Variables	*p*
Demographics	
Age	.1047
Gender	.3237
Race	.7887
Highest education level achieved	.3130
Annual household income	.0389
Marital status	.8490
Legal status in state of residence	.2387
Frequency of use (days per month)	.0169
Duration of use	.0344
Known amount of CBD per day	.0137
Known amount of THC per day	.5379

*p* < .05 determined to be statistically significant

### Stigma

Reported stigma scores by domain and total are reported in Table 6. With six items in each domain, individual item ratings were scored 1–5 points on a Likert-style scale, and each stigma domain score range was 6–30 points. Higher scores correlate with greater stigma experiences. Among the four stigma domains, anticipated stigma scores were the highest (mean = 14.80 ± 7.06). The most significant concerns related to anticipated stigma were that healthcare providers would treat patients differently in the future based on their cannabis use history (mean = 2.67 ± 1.35), not listen to their concerns (mean = 2.56 ± 1.28) or look down on them (mean = 2.52 ± 1.33). Enacted stigma scores were the second highest among the four domains (mean = 12.58 ± 6.78). Within this domain, respondents primarily indicated that healthcare workers have treated them differently based on knowledge of their cannabis use (mean = 2.26 ± 1.27). Perceived stigma was the third most experienced among the four domains (mean = 12.09 ± 5.87). Within perceived stigma, participants most strongly indicated feelings that healthcare workers without a cannabis use history could never really understand them (mean = 2.61 ± 1.31). This is consistent with findings in the Hagani et al. [22] study in which prior personal cannabis use was associated with more positive attitudes. Internalized stigma was the least encountered stigmatization experience captured among the four domains (mean = 8.62 ± 1.27). A complete item analysis for stigma is included in Additional file 3.

**Table 6:** Stigma scores and significance for frequency of cannabis use disclosure

Domain	Minimum	Maximum	Mean ± SD	*p*
Perceived stigma	6	30	12.09 ± 5.87	.0652
Anticipated stigma	6	30	14.80 ± 7.06	.0015
Internalized stigma	6	30	8.62 ± 4.37	.1462
Enacted stigma	6	30	12.58 ± 6.78	.4566
Total Stigma	24	120	48.09 ± 19.91	.0489

*N* = 234, Wald Chi-square test utilized to analyze effects of stigma on the frequency of cannabis use disclosure, *p* < .05 determined to be statistically significant

There was no statistically significant association detected between the frequency of cannabis use disclosure and internalized, enacted, or perceived stigma categories when measured individually. Higher anticipated stigma scores, though, were strongly correlated with participants indicating they never disclosed their history of cannabis use [95% CI 1.045–1.164; *p* = .0015]. A higher overall stigma score was also strongly correlated with never declaring cannabis use [95% CI 1.001–1.039] or only sometimes disclosing [95% CI 1.001–1.035].

## Discussion

The reported cannabis use characteristics align closely with national trends, mirroring prevalent reasons for usage, such as anxiety and pain [14, 20]. Notably, the stigma surrounding these conditions may impact study outcomes [28, 47]. The potential role of anxiety as a predictive risk or protective factor in problematic cannabis use remains uncertain [26]. Risks associated with cannabis use span psychoses, cardiometabolic derangements, respiratory disease, and others [12, 26]. While cannabis is employed as a treatment modality, its efficacy often complements rather than replaces conventional therapies, as seen in opioid-sparing for pain management [21]. Effective prioritization should encompass both cannabinoid profiles and dose stratification. Despite the lack of federal regulation, a substantial proportion of participants (78.7%, 196/249) were aware of the CBD or THC doses in consumed products. The known average THC concentration used exceeded 15% for most (60.5%, 118/195), which is classified as highly potent [50]. Furthermore, a noteworthy 33.1% (82/248) reported cannabis use at least three times daily, potentially correlating with problematic usage patterns and reinforcing stigmatization concerns [51].

Despite varied experiences, participants exhibited low average stigma scores, an important finding to be interpreted alongside the prevalence of fully legal cannabis access (61%, 152/249). Only 1.2% (3/249) of respondents reported living in a state in which cannabis is fully illegal. Most participants reported frequent and prolonged cannabis use (more than five years; 65.7%, 163/248). The highest earning category was selected for in the annual household income in the largest proportion of respondents (35.7%, 89/249). Legalization, frequency, and chronicity of cannabis use, as well as higher income categories, have all been historically associated with decreased stigma experiences [27, 28, 33, 35]. This study lends further support to those trends.

This study also underscores a significant gap in healthcare providers’ recognition of cannabis use, with only 15.1% initiating discussions. This deficit poses potential risks such as unacknowledged medication interactions, unidentified medical issues, and disruptions to emotional well-being. As comfort level with the healthcare provider was reported as being the most important influencer in the desire to disclose cannabis use, inference can be made that discomfort persists for patients. The patient–provider relationship should be upheld as an intimate one in which the essence of knowledge sharing leads to the best care outcomes. Information sharing surrounding cannabis use, however, is a newer concept, as is reliably assessing for cannabis use and applying principles to the plan of care. A gap in the ability to initiate related conversations and overcome stigma persists.

This study aligns with prior research indicating poor communication between patients and primary care providers regarding cannabis use [14]. There is a lack of awareness among providers, both in our study, with a 57.1% occurrence in patient-initiated cannabis use disclosure, and Kondrad et al. [14], with primary care providers unaware of cannabis use 53% of the time. These results accentuate the importance of regular cannabis screening. Failure of the provider to examine and failure of the patient to disclose cannabis use history undoubtedly leads to deficits in knowledgeable care provision. Following this reasoning, recently developed guidelines have called for regular cannabis screening of all patients [52]. Our study, combined with prior research, indicates that despite the perceived wisdom of utilizing such information for treatment decisions, stigmatization, particularly anticipated stigma, may significantly influence the accuracy of cannabis use disclosure during the screening process [40]. Lack of patient comfort with the provider may further impair accuracy and limit the ability to have a clear conversation about cannabis use. Providers must be openly aware of these points to preempt failures or breakdowns in routine screening practices, preceding failures to address worsening medical conditions for which cannabis is used as a treatment modality, drug interactions, and adverse effects, among others. Proposed stigma reduction strategies, including educational programs and interpersonal contact, may address this issue [53]. However, further research is needed to gauge the impact of these interventions on both anticipated stigma and cannabis-related outcomes. Routine screening should inform the plan of care and is crucial in procedural settings, given that cannabis users may present heightened risks [12]. Without securing preoperative knowledge of cannabis use through an unbiased and comprehensive assessment, providers cannot readily employ evidence-based strategies that mitigate associated risks. Yet, further research is needed to determine the impact of routine screening practices on treatment-seeking behaviors and outcomes. For example, as patients may anticipate a medical procedure could be delayed or canceled with provider knowledge of recent cannabis use, impacts on disclosure should be further explored.

Aside from comfort level with the healthcare provider, common themes were revealed among participants in their reasoning for disclosing cannabis use, chiefly citing concerns about potential drug interactions. Some indicated a desire to substitute cannabis for traditional pain medications and for medical records to be complete. A secondary theme was transparency fostered in the provider–patient relationship through disclosure, with participants stating they “want the provider to have the full story” and desiring “full transparency.” Participants furthermore demonstrated self-efficacy to inform their provider about cannabis use, indicating a “need to break the stigma” and unashamedly advocating for themselves. When doing so, however, this can often be met with a knowledge deficit on behalf of the provider and, subsequently, an inability to carry out a productive conversation about cannabis [54]. A resulting, major theme influencing disclosure was also the desire to educate providers about cannabis, with participants stating, “I want them to know so they can provide better care,” but also that “they are too judgmental.” Participants expressed providers would be surprised to learn of cannabis use prevalence among their patients, a need to depart from implying associated drug abuse, and to achieve “accurate medical care.” Undoubtedly, healthcare providers are responsible for providing care through evidence-based interventions. This encompasses a deeper scientific understanding of the endocannabinoid system, pharmacokinetics and pharmacodynamics of cannabis, and related drug interactions [8]. Evidence-based recommendations are limited but should begin with assessment accuracy and the ability to initiate an unbiased and therapeutic conversation. Stigma remains a powerful barrier in treatment-seeking and utilization. Cannabis use screening is an important intervention but must be better utilized alongside a knowledge set in cannabis pharmacology. Stigma-reducing initiatives in screening practices are much needed to better promote patient care [40]. Anticipated stigma, which is based on worry about how one may be perceived if cannabis use were known, postulates priority consideration as mean scores were highest for this domain, and this has been associated with worsened emotional and physical health outcomes [46].

### Limitations

Demographic data for this study revealed a homogenous sample population of small size; therefore, results may not be made generalizable. Descriptive data revealed that most participants were middle-aged white, married, and highly educated females. The average household income was more than $105,000 for most participants surveyed. These results likely reflect the target demographics served by our primary survey distributors. Additionally, since these organizations have common affiliations with nurses, survey respondents included nurses and other healthcare professionals, which may have influenced the results. Although healthcare professionals themselves can undoubtedly provide the perspective of being a patient at times, future studies should seek to distinguish primary occupation clearly.

## Conclusion

Discussions surrounding cannabis use within the patient–provider relationship are most often initiated by the patient and depend largely on the comfort level with the healthcare provider. The results of this study should be used to inform the development and validation of a new cannabis-specific stigma scale. Screening for cannabis use is important but should be conducted in an unbiased manner. Special consideration should be paid to the role anticipated stigma has in influencing the frequency of cannabis use disclosure. Further education of healthcare providers is needed to decrease cannabis-related stigma, promote transparent conversations, and improve assessment practices for patients in the healthcare setting.

### Supplementary Information

**Additional file 1.** Complete Survey.

**Additional file 2.** Frequency of Cannabis Use Disclosure with Healthcare Provider by Variable.

**Additional file 3.** Descriptive Statistics for Each Stigma Item.

## Data Availability

All quantitative data generated or analyzed during this study are included in this published article and its supplementary information files. Qualitative datasets used and/or analyzed during the current study are available from the corresponding author on reasonable request.

## Abbreviations

US: United States
CBD: Cannabidiol
THC: Tetrahydrocannabinol
PCP: Primary care provider
HIV: Human immunodeficiency virus
AIDS: Acquired immunodeficiency syndrome
SU-SMS: Substance Use Stigma Mechanisms Scale
SASSS: Substance Abuse Self-Stigma Scale
