# Supplementary material for: The role of stigma in cannabis use disclosure: an exploratory study
Source: Harm Reduct J. 2024 Jan 26;21:21. doi: 10.1186/s12954-024-00929-8 (PMC10811895; doi:10.1186/s12954-024-00929-8)
Supplement: Supplementary file 1 — Additional file 1. Complete Survey. [file 12954_2024_929_MOESM1_ESM.docx]

Disclosure Patterns and Stigmatization Among Cannabis Users in the U.S. Healthcare System

Start of Block: Info

**Exempt Study Participant Information Sheet**
 **Northeastern University, Department of:** Nursing
 **Name of Investigator(s):** Daniel D. King, DNP, CRNA, CPPS; Carey S. Clark, PhD, RN, AHN-BC, RYT, FAAN; Christopher J. Gill, PhD, MBA, CRNA
 **Title of Project:** Evaluation of Disclosure Patterns and Stigmatization Among Cannabis Users in the U.S. Healthcare System

 **Request to Participate in Research**
 We would like to invite you to take part in a research project. The purpose of this research is to assess for rates and associations of cannabis use disclosure and discussion with consideration for stigmatization as patients in the healthcare system.

 **You must be at least 21 years old to be in this research project.**

 If you decide to take part in this study, we will ask you fill out an online survey of demographic characteristics, cannabis use characteristics, disclosure patterns, and assessment of stigma domains that will take approximately 10 minutes. The survey will be immediately available to you when you consent to participate.

 **The possible risks or discomforts of the study are minimal.**You may feel a little uncomfortable answering personal questions. You may exit the study at any time.

 **You may be eligible for an incentive at the completion of this study that may include discounts on products or a raffle drawing for an item not to exceed $200 value.** Additionally, your answers may help us to learn more about what educational topics, materials, and/or methods, will improve healthcare provider knowledge, potentially reduce stigmatization, and enhance their clinical application of the knowledge. Your part in this study will be handled in a confidential manner. Only the researchers will know that you participated in this study. Any reports or publications based on this research will use only group data and will not identify you or any individual as being of this project.

 **Your part in this study is anonymous.**That means no one will know if you took part in this study and no one, including the researchers, will know what your answers are. Any reports or publications based on this research will use only group data and will not identify you or any individual as being of this project. 

 **It is possible that respondents could be identified by the IP address or other electronic record associated with the response. Neither the researcher nor anyone involved with this survey will be capturing those data.** If you have any questions regarding electronic privacy, please feel free to contact Northeastern University’s Office of Information Security via phone at 617-373-7901, or via email at privacy@neu.edu.

 **The decision to participate in this research project is up to you.** You do not have to participate if you do not want to. Even if you begin the study, you can refuse to answer any question and you may withdraw at any time.

 **If you have any questions about this study**, please feel free to contact Daniel King, Principal Investigator, Email: da.king@northeastern.edu. If you have any questions about your rights in this research, you may contact Nan C. Regina, Director, Human Subject Research Protection, Mail Stop: 560-177, 360 Huntington Avenue, Northeastern University, Boston, MA 02115. Tel: 617.373.4588, Email: n.regina@northeastern.edu. You may call anonymously if you wish.

 **This study has been reviewed and approved by the Northeastern University Institutional Review Board (# 22-06-17).

 By clicking on the “accept” button below you are indicating that you consent to participate in this study. Please print out a copy of this consent screen or download a copy of the consent form for your records.**

 Thank you.

 *Daniel King, DNP, CRNA, CPPS*

End of Block: Info

Start of Block: Inclusion/Exclusion

Throughout this survey, the term **cannabis** refers to *any* product derived from the cannabis plant including, but not limited to flowers, buds, oils, tinctures, concentrates, extractions, and edibles. The term cannabis includes marijuana and hemp. Cannabis contains many cannabinoids or chemical compounds including, but not limited to, delta-9 tetrahydrocannabinol (THC), cannabidiol (CBD), and cannabinol (CBN).

Have you used cannabis within the last 5 years?

- Yes
- No

Have you received medical care within the last 5 years?

- Yes
- No/Do Not Recall

Are you 21 years or older?

- Yes
- No

Display This Question:

If Have you used cannabis within the last 5 years? = No

Or Have you received medical care within the last 5 years? = No/Do Not Recall

Or Are you 21 years or older? = No

***You answered "no" to one of the previous questions, indicating that you do not meet the requirements to be included in this study. If this is not the case, please use the back arrow to adjust your responses now. If this is in fact the case, you should exit the survey by clicking X in the upper right-hand corner at this time. Thank you for volunteering!***

End of Block: Inclusion/Exclusion

Start of Block: Demographics

Please enter your age (in years):

________________________________________________________________

What is your gender?

- Male
- Female
- Transgender Male
- Transgender Female
- Nonbinary
- Gender variant/Non-conforming
- Prefer not to answer
- Other (please specify): __________________________________________________

What is your race?

- White
- Black or African American
- American Indian or Alaska Native
- Asian
- Native Hawaiian or Pacific Islander
- Prefer not to answer
- Other (please specify): __________________________________________________

In which state do you currently reside?

▼ Alabama ... I do not reside in the United States

What is your HIGHEST level of education?

- Less than high school/GED
- High school/GED
- Technical school
- Associate degree
- Bachelor's degree
- Master's degree
- Doctoral degree
- Prefer not to answer

What is your annual household income? *(Household income is defined as all cash received before taxes (i.e., wage, salary, pension, social security), received within a 12-month period by all members of a household 15 years and older)*

- Less than $35,000
- $35,000 to less than $70,000
- $70,000 to less than $105,000
- $105,000 or more
- Prefer not to answer

What is your marital status?

- Now married
- Widowed
- Separated/Divorced
- Never married
- Prefer not to answer

End of Block: Demographics

Start of Block: Cannabis Use Patterns

What route do you MOST often use to consume cannabis?

- Capsule
- Edible
- Gum
- Lozenge
- Oil
- Smoking
- Spray
- Tincture
- Vape
- Suppository
- Topical
- Other (please specify): __________________________________________________

Do you know the amount(s)/ mg of cannabidiol (CBD) and/or tetrahydrocannabinol (THC) contained in products you normally consume?

- Yes
- No

Display This Question:

If Do you know the amount(s)/ mg of cannabidiol (CBD) and/or tetrahydrocannabinol (THC) contained in... = Yes

What is the average amount of cannabidiol (CBD) you normally consume?

- 5-20 mg/day
- 21-29 mg/day
- more than 30 mg/day
- unknown

Display This Question:

If Do you know the amount(s)/ mg of cannabidiol (CBD) and/or tetrahydrocannabinol (THC) contained in... = Yes

What is the average amount of tetrahydrocannabinol (THC) you normally consume?

- 1-5 mg/day
- 5-10 mg/day
- 10-20 mg/day
- 20-30 mg/day
- more than 30 mg/day
- Unknown

Display This Question:

If Do you know the amount(s)/ mg of cannabidiol (CBD) and/or tetrahydrocannabinol (THC) contained in... = Yes

What concentration of THC (tetrahydrocannabinol) do you normally consume?

- Less than 2.49%
- 2.5 – 9.99%
- 10-14.99%
- 15-19.99%
- 20-24.99%
- Greater than 25%
- Unknown

During the last 30 days, how many days did you use cannabis?

- 1 day or less
- 2-5 days
- 5-10 days
- 11-20 days
- 21 days or more

On the days you used cannabis, how often did you use cannabis?

- Once
- Twice
- Three times
- Greater than three times
- Unknown/do not recall

How long have you been using cannabis?

- Less than 1 year
- 1-5 years
- 5-10 years
- Greater than 10 years

For what reason(s) do you use cannabis? **(Select all that apply)**

- Anxiety
- Appetite
- Arthritis
- Autoimmune disease
- Brain disorder
- Bladder disorder
- Bowel disease
- Cancer
- Depression
- Glaucoma
- Headache/migraine
- Hepatitis
- HIV/AIDS
- Kidney disease
- Muscle spasm
- Neuromuscular disease
- Neuropathy
- Nausea/vomiting
- Pain
- Recreation/leisure
- Seizure
- Spinal cord disease
- Post-traumatic stress disorder
- Sleep
- Terminal illness
- Other(s) (please specify): __________________________________________________

End of Block: Cannabis Use Patterns

Start of Block: Cannabis Disclosure

When in the healthcare setting, who initiates discussion of your cannabis use?

- Myself
- Healthcare provider
- Neither myself nor healthcare provider

How often do you make your cannabis usage known to healthcare providers?

- Always
- Sometimes
- Never

What most influences your desire to disclose your cannabis use?

- Healthcare provider asks
- Comfort level with healthcare provider
- Unknown
- I do not disclose my cannabis use
- Other (please specify: __________________________________________________

End of Block: Cannabis Disclosure

Start of Block: Stigma Domains

**How often have healthcare workers treated you this way in the past because of your cannabis use history?**

|  | Never | Not often | Somewhat | Often | Very often |
| --- | --- | --- | --- | --- | --- |
| Healthcare workers have thought I cannot be trusted. |  |  |  |  |  |
| Healthcare workers have looked down on me. |  |  |  |  |  |
| Healthcare workers have treated me differently. |  |  |  |  |  |
| Healthcare workers have not listened to my concerns. |  |  |  |  |  |
| Healthcare workers have thought that I’m pill shopping, or trying to con them into giving me prescription medications to get high or sell. |  |  |  |  |  |
| Healthcare workers have given me poor care. |  |  |  |  |  |

**How do you feel about your cannabis use history?**

|  | Strongly disagree | Disagree | Neither disagree nor agree | Agree | Strongly agree |
| --- | --- | --- | --- | --- | --- |
| Having used cannabis makes me feel like I’m a bad person. |  |  |  |  |  |
| I feel I’m not as good as others because I used cannabis. |  |  |  |  |  |
| I feel ashamed of having used cannabis. |  |  |  |  |  |
| I think less of myself because I used cannabis. |  |  |  |  |  |
| Having used cannabis makes me feel unclean. |  |  |  |  |  |
| Having used cannabis is disgusting to me. |  |  |  |  |  |

**How likely is it that healthcare workers will treat you this way *in the future* because of your cannabis use history?**

|  | Very unlikely | Unlikely | Neither unlikely nor likely | Likely | Very likely |
| --- | --- | --- | --- | --- | --- |
| Healthcare workers will think that I cannot be trusted. |  |  |  |  |  |
| Healthcare workers will look down on me. |  |  |  |  |  |
| Healthcare workers will treat me differently. |  |  |  |  |  |
| Healthcare workers will not listen to my concerns. |  |  |  |  |  |
| Healthcare workers will think that I’m pill shopping, or trying to con them into giving me prescription medications to get high or sell. |  |  |  |  |  |
| Healthcare workers will give me poor care. |  |  |  |  |  |

**Please read each statement below and select the number that indicates how many people you think would react to you as described. Please use the scale below, and please do not omit any item.**

|  | Few people (0-20%) | Some people (20-40%) | Many people (40-60%) | Most people (60-80%) | Almost everyone (80-100%) |
| --- | --- | --- | --- | --- | --- |
| Healthcare workers will think I’m worthless if they know about my cannabis use history. |  |  |  |  |  |
| Healthcare workers without a cannabis use history could never really understand me. |  |  |  |  |  |
| If healthcare workers were to find out about my history of cannabis use, they would expect me to be weak-willed. |  |  |  |  |  |
| Healthcare workers would be scared of me if they knew about my cannabis use history. |  |  |  |  |  |
| If healthcare workers were to find out about my history of cannabis use, they would doubt my character. |  |  |  |  |  |
| Healthcare workers will think I have little talent or skill if they know about my cannabis use history. |  |  |  |  |  |

End of Block: Stigma Domains
