# Supplementary material for: The role of stigma in cannabis use disclosure: an exploratory study
Source: Harm Reduct J. 2024 Jan 26;21:21. doi: 10.1186/s12954-024-00929-8 (PMC10811895; doi:10.1186/s12954-024-00929-8)
Supplement: Supplementary file 2 — Additional file 2. Frequency of Cannabis Use Disclosure with Healthcare Provider by Variable. [file 12954_2024_929_MOESM2_ESM.docx]

| **Supplement 2. Frequency of Cannabis Use Disclosure with Healthcare Provider by Variable** | | | | |
| --- | --- | --- | --- | --- |
| Variable | Frequency of Cannabis Use Disclosure | | | *P (Test)* |
|  | Always | Sometimes | Never |  |
| Age | | | | |
| < 38 years | 15 (7.11%) | 21 (9.95%) | 12 (5.69%) | .1047 (Chi-square) |
| 38-61 years | 50 (23.70%) | 29 (13.74%) | 25 (11.85%) |  |
| > 61 years | 33 (15.64%) | 15 (7.11%) | 11 (5.21%) |  |
| Gender | | | | |
| Female | 66 (31.28%) | 45 (21.33%) | 38 (18.01%) | .3237 (Chi-square) |
| Male | 32 (15.17%) | 20 (9.48%) | 10 (4.74%) |  |
| Race | | | | |
| White | 82 (38.86%) | 54 (25.59%) | 42 (19.91%) | .7887 (Chi-square) |
| Black or African American | 16 (7.58%) | 11 (5.21%) | 6 (2.84%) |  |
| Cannabis Legalization Status in Reported State of Residence | | | | |
| Legalized | 65 (31.1%) | 41 (19.62%) | 23 (11.00%) | .2387(Fisher’s Exact) |
| Medical and decriminalized | 11 (5.26%) | 12 (5.74%) | 10 (4.78%) |  |
| Medical | 14 (6.70%) | 8 (3.83%) | 8 (3.83%) |  |
| CBD with THC as an ingredient only | 2 (0.96%) | 0 (0.00%) | 3 (1.44%) |  |
| Decriminalized | 2 (0.96%) | 0 (0.00%) | 3 (1.44%) |  |
| Fully illegal | 0 (0.00%) | 1 (0.48%) | 1 (0.48%) |  |
| Highest Level of Education | | | | |
| Less than Undergraduate | 44 (20.85%) | 23 (10.90%) | 14 (6.64%) | .3130 (Chi-square) |
| Bachelors | 31 (14.69%) | 21 (9.95%) | 16 (7.58%) |  |
| Graduate | 23 (10.9%) | 21 (9.95%) | 18 (8.53%) |  |
| Annual Household Income | | | | |
| < $35,000 | 28 (13.27%) | 14 (6.64%) | 9 (4.27%) | .0389 (Chi-square) |
| $35,000 to less than $70,000 | 22 (10.43%) | 9 (4.27%) | 4 (1.90%) |  |
| $70,000 to less than $105,000 | 20 (9.48%) | 14 (6.64%) | 7 (3.32%) |  |
| > $105,000 | 24 (11.37%) | 26 (12.32%) | 27 (12.80%) |  |
| Prefer not to answer | 4 (1.90%) | 2 (0.95%) | 1 (0.47%) |  |
| Marital Status | | | | |
| Now married | 49 (23.22%) | 37 (17.54%) | 26 (12.32%) | .8490 (Chi-square) |
| Widowed/separated/divorced | 30 (14.22%) | 15 (7.11%) | 14 (6.64%) |  |
| Never married = 44 (17.7%) | 19 (9.00%) | 13 (6.16%) | 8 (3.79%) |  |
| Cannabis use frequency in last 30 days | | | | |
| 1 day or less | 4 (1.90%) | 3 (1.42%) | 2 (0.95%) | .2387 (Fisher’s Exact) |
| 2-5 days | 3 (1.42%) | 7 (3.32%) | 6 (2.84%) |  |
| 5-10 days | 2 (0.95%) | 7 (3.32%) | 5 (2.37%) |  |
| 11-20 days | 7 (3.32%) | 5 (2.37%) | 7 (3.32%) |  |
| 21 days or more | 82 (38.86%) | 43 (20.38%) | 28 (13.27%) |  |
| Duration of use | | | | |
| Less than 1 year | 5 (2.37%) | 3 (1.42%) | 8 (3.79%) | .0344 (Chi-square) |
| 1-5 years | 22 (10.43%) | 14 (6.64%) | 17 (8.06%) |  |
| 5-10 years | 19 (9.00%) | 13 (6.16%) | 4 (1.90%) |  |
| Greater than 10 years | 52 (24.64%) | 35 (16.59%) | 19 (9.00%) |  |
| Average Amount of CBD Known | | | | |
| 5-20 mg/day | 42 (25.30%) | 29 (17.47%) | 22 (13.25%) | .0137 (Chi-square) |
| 21-29 mg/day | 16 (9.64%) | 3 (1.81%) | 5 (3.01%) |  |
| > 30 mg/day | 19 (11.45%) | 6 (3.61%) | 2 (1.20%) |  |
| Unknown | 5 (3.01%) | 10 (6.02%) | 7 (4.22%) |  |
| Average Amount of THC Known | | | | |
| 1-5 mg/day | 12 (7.23%) | 11 (6.63%) | 8 (4.82%) | .5379 (Chi-square) |
| 5-10 mg/day | 11 (6.63%) | 6 (3.61%) | 5 (3.01%) |  |
| 10-20 mg/day | 16 (9.64%) | 10 (6.02%) | 9 (5.42%) |  |
| 20-30 mg/day | 19 (11.45%) | 6 (3.61%) | 5 (3.01%) |  |
| > 30 mg/day | 18 (10.84%) | 9 (5.42%) | 3 (1.81%) |  |
| Unknown | 6 (3.61%) | 6 (3.61%) | 6 (3.61%) |  |
| ***P*< .05 determined to be statistically significant** | | | | |
