# Supplementary material for: The role of stigma in cannabis use disclosure: an exploratory study
Source: Harm Reduct J. 2024 Jan 26;21:21. doi: 10.1186/s12954-024-00929-8 (PMC10811895; doi:10.1186/s12954-024-00929-8)
Supplement: Supplementary file 3 — Additional file 3. Descriptive Statistics for Each Stigma Item. [file 12954_2024_929_MOESM3_ESM.docx]

| **Supplement 3. Descriptive statistics for each stigma item (n = 234)** | | |
| --- | --- | --- |
| Stigma Domain | Mean | SD |
| Perceived | | |
| Please read each statement below and circle the number that indicates how many people you think would react to you as described. Please use the scale below, and please do not omit any item. *(1=few people (0-20%), 2=some people (20-40%), 3=many people (40-60%), 4=most people (60-80%), 5=almost everyone (80-100%))* |  | |
| Healthcare workers will think I’m worthless if they know about my cannabis use history. | 1.85 | 1.10 |
| Healthcare workers without a cannabis use history could never really understand me. | 2.61 | 1.31 |
| If healthcare workers were to find out about my history of cannabis use, they would expect me to be weak-willed | 1.94 | 1.15 |
| Healthcare workers would be scared of me if they knew about my cannabis use history. | 1.54 | 0.88 |
| If healthcare workers were to find out about my history of cannabis use, they would doubt my character. | 2.19 | 1.23 |
| Healthcare workers will think I have little talent or skill if they know about my cannabis use history. | 1.97 | 1.15 |
| Anticipated | | |
| How likely is it that healthcare workers will treat you this way in the future because of your cannabis use history? *(1=very unlikely, 2=unlikely, 3=neither unlikely nor likely, 4=likely, 5=very likely)* |  | |
| Healthcare workers will think that I cannot be trusted. | 2.39 | 1.25 |
| Healthcare workers will look down on me. | 2.52 | 1.33 |
| Healthcare workers will treat me differently. | 2.67 | 1.35 |
| Healthcare workers will not listen to my concerns. | 2.56 | 1.28 |
| Healthcare workers will think that I’m pill shopping, or trying to con them into giving me prescription medications to get high or sell. | 2.29 | 1.34 |
| Healthcare workers will give me poor care. | 2.38 | 1.23 |
| Internalized | | |
| How do you feel about your cannabis use history? *(1=strongly disagree, 2=disagree, 3=neither disagree nor agree, 4=agree, 5=strongly agree)* |  | |
| Having used cannabis makes me feel like I’m a bad person. | 1.55 | 0.96 |
| I feel I’m not as good as others because I used cannabis. | 1.53 | 0.99 |
| I feel ashamed of having used cannabis. | 1.52 | 0.93 |
| I think less of myself because I used cannabis. | 1.46 | 0.90 |
| Having used cannabis makes me feel unclean. | 1.37 | 0.76 |
| Having used cannabis is disgusting to me. | 1.20 | 0.50 |
| Enacted | | |
| How often have healthcare workers treated you this way in the past because of your cannabis use history? *(1=never, 2=not often, 3=somewhat often, 4=often, 5=very often)* |  | |
| Healthcare workers have thought I cannot be trusted. | 1.93 | 1.18 |
| Healthcare workers have looked down on me. | 2.24 | 1.30 |
| Healthcare workers have treated me differently. | 2.26 | 1.27 |
| Healthcare workers have not listened to my concerns. | 2.22 | 1.24 |
| Healthcare workers have thought that I’m pill shopping, or trying to con them into giving me prescription medications to get high or sell. | 1.84 | 1.31 |
| Healthcare workers have given me poor care. | 2.11 | 1.24 |
